# PERSONALITY BYTES: CRACKING THE CODE OF OLDER ADULTS ENGAGEMENT WITH AI

**DOI:** 10.1093/geroni/igae098.1905

**Published:** 2024-12-31

**Authors:** Marcia Shade, Changmin Yan, Valerie Jones, Julie Boron

**Affiliations:** University of Nebraska Medical Center, Omaha, Nebraska, United States; University of Nebraska Lincoln, Lincoln, Nebraska, United States; University of Nebraska Lincoln, Lincoln, Nebraska, United States; University of Nebraska Omaha, Omaha, Nebraska, United States

## Abstract

Despite stereotypes about older adults’ reluctance to embrace technology, smartphone and technological device adoption among this demographic is increasing, including the use of Artificial Intelligence (AI). However, perceptions about AI may hinder engagement in research among older adults. This study examined how older adults’ personalities influence their interaction with AI-driven interactive routines. Fifty community-dwelling older adults participated in a 12-week randomized pilot study, divided into standard and enhanced groups, interacting with conversational AI for intervention routines. Data collected included personality traits, usability, and routine initiations. Participants, averaging 72.4 years old, were mostly female (88%) and White (95%). Personality traits did not significantly affect routine initiations overall, but extraversion predicted engagement throughout the day and morning in both groups. Evening routines were unaffected by personality traits. Agreeableness and conscientiousness significantly predicted perceived usability. These findings emphasize the influence of older adults’ personality traits on AI technology interaction, with extraversion playing a significant role. Additionally, agreeableness and conscientiousness impact usability. Integrating personality traits into research protocols involving technology interactions is crucial for better understanding older adults’ engagement.

